# Why do marital partners of people living with HIV not test for HIV? A qualitative study in Lusaka, Zambia

**DOI:** 10.1186/s12889-016-3396-z

**Published:** 2016-08-25

**Authors:** Maurice Musheke, Sonja Merten, Virginia Bond

**Affiliations:** 1 Population Council, Private Bag RW319x, Lusaka, Zambia; 2 Swiss Tropical and Public Health Institute, Socinstrasse 57, CH-4002 Basel, Switzerland; 3 University of Basel, Petersplatz 1, CH-4003 Basel, Switzerland; 4 Zambart Project, University of Zambia, P.O. Box 50697, Lusaka, Zambia; 5 Department of Global Health and Development, Faculty of Public Health and Policy, London School of Hygiene and Tropical Medicine, Keppel Street, London, WC1E 7HT UK

**Keywords:** HIV, HIV testing, Marital partner, Antiretroviral therapy, Couple counselling, Zambia

## Abstract

**Background:**

Knowledge of HIV status is crucial for HIV prevention and management in marital relationships. Yet some marital partners of people living with HIV decline HIV testing despite knowing the HIV-positive status of their partners. To date, little research has explored the reasons for this.

**Methods:**

An exploratory qualitative study was undertaken in Lusaka, Zambia, between March 2010 and September 2011, nested within a larger ethnographic study. In-depth interviews were held with individuals who knew the HIV-positive status of their marital partners but never sought HIV testing (*n* = 30) and HIV service providers of a public sector clinic (*n* = 10). A focus group discussion was also conducted with eight (8) lay HIV counsellors. Data was transcribed, coded and managed using ATLAS.ti and analysed using latent content analysis.

**Results:**

The overarching barrier to uptake of HIV testing was study participants’ perception of their physical health, reinforced by uptake of herbal remedies and conventional non-HIV medication to mitigate perceived HIV-related symptoms. They indicated willingness to test for HIV if they noticed a decline in physical health and other alternative forms of care became ineffective. Also, some study participants viewed themselves as already infected with HIV on account of the HIV-positive status of their marital partners, with some opting for faith healing to get ‘cured’. Other barriers were the perceived psychological burden of living with HIV, modulated by lay belief that knowledge of HIV-positive status led to rapid physical deterioration of health. Perceived inability to sustain uptake of life-long treatment – influenced by a negative attitude towards treatment – further undermined uptake of HIV testing. Self-stigma, which manifested itself through fear of blame and a need to maintain moral credibility in marital relationships, also undermined uptake of HIV testing.

**Conclusions:**

Improving uptake of HIV testing requires a multi-pronged approach that addresses self-stigma, lay risk perceptions, negative treatment and health beliefs and the perceived psychological burden of living with HIV. Strengthening couple HIV testing services, including addressing conflict and addressing gendered power relationships are also warranted to facilitate joint knowledge, acceptance and management of HIV status in marital relationships.

## Background

A 2016 Joint United Nations Programme on AIDS (UNAIDS) report indicates that half of all people living with HIV (PLHIV) are unaware of their HIV status [1]. In sub-Saharan Africa (SSA), which has a generalized HIV epidemic, most HIV infections occur in marital or cohabiting relationships [2–4], for instance, 50–65 % in Swaziland, 35–62 % in Lesotho and 44 % in Kenya [4]. According to data from 27 cohorts totaling 13,061 sero-discordant couples in SSA, and Demographic and Health Survey (DHS) data on 1,145 sero-discordant couples in 14 countries, the proportion of HIV-positive women in stable heterosexual sero-discordant relationships was 47 % [5].

Despite the progressive roll-out of different HIV testing initiatives in many settings of SSA as a crucial HIV prevention strategy, barriers to HIV testing have persisted. These barriers include stigma and discrimination [5, 6], self-perception of being at less risk of infection [7–9], perceived inability by service providers to maintain confidentiality [8, 10, 11], lack of symptoms or deterioration of health [12–14] and women’s lack of control over decisions related to HIV testing [15–17].

Zambia has an estimated HIV prevalence of 13.3 % in the adult population aged 15–49 years [18]. The HIV prevalence peaked in the late 1990s before levelling off and declining to current rates [19]. For marital partners, the need to test as an HIV prevention and management strategy is even more critical. Empirical data on urban Zambia shows that at least 60 % of new heterosexual HIV infections occur within marriages or cohabiting relationships [20], and among married/cohabiting partners, discordance rate is about 11 % [18]. HIV testing by marital partners is thus critical to ensure: increased uptake and adherence to treatment [5]; increased uptake and adherence to HIV treatment for own health (which in turn decreases drug resistance, morbidity and mortality); adoption of risk reduction sexual behaviour [5, 21, 22]; decreased stigma and normalization [5]; and uptake of treatment for prevention of mother-to-child transmission of HIV by women [5, 23, 24].

While a recent systematic review has synthesised the factors influencing uptake of HIV testing in SSA [25], there is still a dearth of information on specifically why individuals aware of the HIV-positive status of their marital partners do not seek HIV testing. Understanding these barriers is critical in the prevention of HIV transmission and management of HIV within marital relationships. Therefore, to contribute to the body of knowledge on barriers to uptake of HIV testing, this study reports the reasons for non-uptake of HIV testing by marital partners of People Living with HIV (PLHIV) who knew the HIV-positive status of their partners.

## Methods

### Study design

This was an exploratory qualitative study nested within a larger 18-month ethnographic study on factors influencing uptake of HIV testing, non-initiation of and retention in ART care. This study design was suitable for identifying and eliciting in-depth insight into factors hindering uptake of HIV testing by individuals who knew the HIV-positive status of their marital partners. In this study, marital partnership referred to a man and women who were officially married whether under statutory or customary law, and were living together as husband and wife.

### Study setting

The study was conducted in a low-income, high-density urban setting, located about 10 km south of the Business District in Lusaka, the capital city of Zambia. Based on field observations, common physical features of the study setting are a crowded mix of formal and informal housing structures, shops and market stalls, one main tarred road with adjoining dusty, unlabeled roads that become water-logged and muddy during the rainy season, and poor solid waste disposal facilities. The majority of the residents have developed both strong kin and non-kin social network relationships. Most of the households are large, in part due to the negative impact of HIV, with some individuals growing up as orphans under the care of extended family members. Although not all family members live together, they still maintain reciprocal social and economic support ties. Other social network relationships are a product of religious affiliations and occupational and social lifestyle activities.

Public social amenities are non-existent in the area. Social life often revolves around spending time in the bars, night clubs and make-shift drinking places; these places often serve as one source of sexual network relationships. Economically, the living conditions of local residents are mixed. Some people are formally employed in government and the private sector. The majority of the people earn their living in the informal sector of the economy, mostly as traders selling fruits, vegetables, meat products, fish, charcoal and second-hand clothes in the city centre markets, other markets within Lusaka, and in the open-air local markets. The unemployment situation is further exacerbated by rural–urban migration, as people move into the city in search of job opportunities and a better life.

Health services are mainly accessed from a public health centre. The health centre has an out-patient unit, an in-patient unit with female and male admission wards, ‘opt-in’ HIV counselling and testing (HCT) unit, an Antiretroviral Therapy (ART) unit, a Maternal and Child Health (MCH) unit which provides antenatal and postnatal health services, and a Tuberculosis (TB) screening and treatment unit. In addition, mobile HCT services in the area are periodically provided by non-governmental HIV service providers. HCT and ART services are provided free of charge. Free couple HCT services are provided at the MCH unit and the opt-in HCT unit of the health centre. The health centre also previously housed a couple HCT project implemented by a local organisation called Zambia Emory HIV research project (ZEHRP). By March 2010, when this qualitative study started, the public health centre had more than 5,000 people on ART and more than 5,000 on pre-ART.

There is also a plethora of privately owned clinics and drug stores. Other health service providers in the area include herbalists, traditional doctors and faith healers, some of whom advertise their services, including ‘cure’ of HIV and sexually transmitted infections (STIs). Tied to healing is Christianity, which is the dominant religion in the area, with a myriad of charismatic evangelical Pentecostal churches, some of which provide faith healing sessions for people suffering from different health conditions, including HIV.

### Participant selection

Marital partners of PLHIV were identified, contacted and recruited through their marital partners receiving ART care at a local health centre. A two stage-recruitment strategy was used to recruit this group of study participants. First, ART clinic staff purposively identified PLHIV who disclosed their status to their marital partners but whose partners opted not to seek HIV testing. Second, PLHIV were then asked to recruit, on behalf of the study, their partners for interviews. Only those spouses who agreed to participate were contacted by the research team to schedule time and location for interviews. Using snowball and opportunistic sampling techniques, health care workers (nurses and lay HCT counsellors) who were involved in the delivery of HCT and ART services were identified and recruited from the various units of the public health centre.

### Data collection and analysis

Data was collected between March 2010 and September 2011 as part of the first author’s doctoral studies in epidemiology. To ensure consistency in the administration of the research tools, all interviews were conducted by the first author, a social scientist with extensive experience in designing and conducting qualitative research. Thirty-eight (38) PLHIV and receiving ART at the local public health center were approached to recruit their marital partners for the study. Thirty-four (34) agreed to talk to and recruit their spouses for the study, out of which thirty (30) participated in open ended, face-to-face, audio-recorded in-depth interviews. The first author lived in the study setting for the entire period of data collection (18 months) and this enabled him to win the trust and confidence of the study participants, thereby enabling them to open up and share their perspectives.

In addition, in-depth interviews were held with health care workers involved in the provision of HCT and ART services (*n* = 10). One focus group discussion (FGD) was also conducted with the health facility-based lay HCT counsellors (*n* = 8). Five (5) of the HCT counsellors were women and the rest were men. No repeat interviews or FGDs were conducted with study participants.

The main research question asked was: “What are the reasons for not seeking HIV testing despite knowing the HIV-positive status of your/their spouses?” Interviews with health care workers and FGD with HCT counsellors were conducted in English while interviews with spouses of PLHIV were conducted in *Nyanja* - the local language mainly spoken in the area. The in-depth interviews lasted between 30 and 45 min and the FGD with HIV counsellors lasted about an hour.

Data collection and preliminary data analysis was a cyclical process. The data collection tools were first piloted and fine-tuned. During actual data collection, interview data informed ensuing interviews and data collection was ended when emerging data became repetitive. All interviews conducted in local language were translated and all interviews were transcribed verbatim. The transcripts were then entered into, and organised and managed using, ATLAS.ti version 6. The data was then coded inductively. The first author developed the coding framework and coded all the data, which were reviewed and approved by the other authors. Team meetings were used to discuss and resolve differences regarding the coding framework and the codes and themes generated.

Qualitative latent content analysis [26] was used to analyse and interpret the data. Latent content analysis involves an analysis of the relationship aspects of the textual data and an interpretation of the underlying meaning of the text, referred to as the latent content [26]. All interview and FGD transcripts constituted our unit of analysis. Unit of analysis refers to all words and phrases of the interview and FGD transcripts [26]. They were read several times to create a sense of the whole data [26, 27].

Within-case and across-case analysis [28] of the interview transcripts was undertaken to inductively generate concepts across the individual interviews. For each interview transcript, we conducted within-case analysis and retrieved and coded reasons for not seeking HIV testing despite knowing the HIV-positive status of a marital partner. Thereafter, we conducted across-case analysis by comparing and contrasting participants’ reasons. Similar codes were then put together to form categories. A category is therefore a group of content that shares a commonality; it is a thread throughout the codes [26]. Themes were then developed by interpreting categories for their underlying meaning. The themes are, therefore, the expression of the latent content (underlying meaning) of the textual data [26]. For instance, codes such as ‘feeling healthy’, ‘not sick’, ‘nothing wrong in the body’ were categorised as ‘state of physical health’ as described in the results section of the paper. The theme generated from this is ‘lay wellness and illness beliefs.’ These themes are described in the discussion section of the paper.

Three reference points were used to identify emergent themes: recurrence, repetition and forcefulness of ideas within the interview data [29]. Through this analytical strategy, we were able to identify themes that cut across study participants but were still grounded in individual perspectives [28]. Lastly, we selected interview and group discussion excerpts that best illustrated these themes. Arising from the rapport and relationships created with study participants during ethnographic fieldwork, emerging findings were shared with study participants.

### Ethical considerations

The study was approved by the Ethics Committee in Basel (Ethik-Kommission beider Basel) and the University of Zambia School of Humanities and Social Sciences Research Ethics Committee, as part of the research project ‘Improving equity of access to HIV care and treatment in Zambia’. Written informed consent was obtained from all study participants. To ensure confidentiality, interviews and FGD with health centre staff took place in private spaces of the health facility while interviews with marital partners of PLHIV took place at locations of their choice. Some preferred to be interviewed at home, in the absence of their spouses; others preferred to be interviewed at neutral locations such as private spaces at public health centre and homes study participants’ friends and relatives.

To protect the identity of study participants, all identifying information was excluded from the interview transcripts. Study participants were given a reimbursement of ZMK50.00 (about US$10 at the time) as compensation for their time. In addition and when appropriate, study participants were provided with refreshments and given transport reimbursement of ZMK50.00 (about US$10 at the time) when interviews were conducted outside their homes.

## Results

### Characteristics of study participants

Half of the marital partners of PLHIV interviewed were women; more than half were aged ≥35 years with the oldest being a 51-year old man. The majority of the study participants were in informal employment; all those in formal employment (*n* = 5) were men. More than half had been married for at least 4 years (Table 1). Two-thirds (*n* = 20) had known the HIV-positive status of their spouses for at least 2 years and all the marital partners of PLHIV were on ART.

**Table 1:** Characteristics of marital partners of PLHIV

Characteristic	Number of Respondents
Age (Years)	
18–24	4
25–34	9
35–44	10
>44	7
Sex	
Male	15
Female	15
Source of livelihood	
Formal employment	5
Informal employment	18
Not working/dependant	7
Duration of marital relationship	
<12 months	0
1–<2 years	1
2–<3 years	7
3–<4 years	4
4–<5 years	10
≥5 years	8
Duration since known HIV status of partner	
6–<12 months	4
1–<2 years	6
2–<3 years	9
3–<4 years	9
≥4 years	2
Treatment status of HIV+ marital partner	
On ART	30
Not on ART	0

The health care workers interviewed belonged to two categories - professional nurses and lay HCT counsellors. The nurses were full-time government employees while lay HCT counsellors worked as volunteers. The lay HCT counsellors, some of whom were living with HIV, worked as HIV testing and treatment supporters. Their responsibilities included providing pre and post HIV counselling, HIV treatment preparedness and adherence counselling, and tracing treatment defaulters (HIV patients not reporting for clinical appointment or pick-up of medication) and bringing them back into care. The nurses and counsellors were all drawn from the HCT, ART, Mother and Child Health (MCH) and TB units of the public health centre.

### Reasons for non-uptake of HIV testing

#### State of physical health

Participants’ own perception of their physical health was the overarching barrier to uptake of HIV testing. They measured good health in terms of functional ability and not the clinical presence of HIV infection. Therefore, poor health was viewed as the sine *qua non* for seeking HIV testing. Asked when they would consider going for HIV testing, one study participant bluntly said: *“[I will test] when I get sick, I mean as in being bed-ridden. Not when I am still strong, I do not see any need to test and I pray that I will not get to that stage”* (30-year old woman). Another study participant replied: “*She [wife] went to the clinic; she tested and was given her results. I saw the results as well*.... *But me, I am just ok*.…*Because I rarely get sick, I do not see any need to go and test. So it is very difficult to just go and test when you feel that there is nothing wrong in your body.”* (47-year old man)

The reluctance to test when still physically strong was echoed by health care providers. A nurse at the ART clinic noted that: *“I think from what I have observed, somebody might know their HIV status but because they still have energy to run around and do their work, they would not care about coming for HIV testing. Most of them wait until they have serious opportunistic infections. That is when we see them rushing here to the clinic.”*

For some men, the perception of being physically healthy could be attributed to the notion of masculinity. They needed to show that they were strong and not reliant on HIV testing and treatment to sustain their physical health. As one male study participant explained: *“When you go and test, when you start taking the drugs, it means you have given up as a man that you are not strong enough on your own; you become drug-dependent, you see what I mean?”* (25-year old man).

Ironically, although some male respondents showed unwillingness to test to show that they could live healthy and normal lives without medication, they still adopted self-care practices of using herbal remedies and conventional non-HIV medication to deal with symptoms of opportunities infections, such as periodic episodes of diarrhoea, rash, sore throats and coughing.

#### Fear of blame by marital partner

Non-uptake of HIV testing was also used to fend off blame or accusations of being responsible for HIV infection. This pattern of blame was most often levelled and articulated by men towards women. It was also often the case in distrustful relationships, in which some study participants blamed their HIV-positive partners for being responsible for HIV infection. Therefore, by not seeking HIV testing, some study participants pointed out that they were able to maintain moral credibility in the marital relationship, that they were not responsible for HIV infection. While a few men suspected their female partners to be responsible for HIV infection, the majority of them acknowledged being the possible source of HIV infection due to extra-marital relationships. Therefore, not testing for HIV was meant to avoid confirming their partners’ suspicions - marital infidelity. One male study participant said: *“If my wife knows that I am HIV-positive, she is going to say “yes, it is because you had other sexual partners”. Sometimes the best way to avoid problems with women is not to test. They cannot blame you for HIV because you have not tested even if deep down your heart, you know that you may be the one who contracted HIV and then infected your wife”* (32-year old man).

For women, and due to power imbalance in marital relationships, they feared that seeking HIV testing could prompt their partners to shift the blame for HIV infection onto them. One female study participant said: *“Sometimes we women fear that if we went for testing, men would take advantage of the situation and shift the blame on us. So to avoid being blamed, some women opt not to go and test.”* (45-year old woman).

These fears were confirmed by lay HCT counsellors and professional health care workers. One lay HCT counsellor said: *“Usually the ones that behave badly are men; when the wife is HIV-positive or even when both are positive, they (men) deny it or claim that they are not responsible for the infection. So women become reluctant to test for HIV for fear of being blamed”* (Female VCT counsellor, FGD).

#### Perception of already being infected with HIV

The majority of the spouses of PLHIV interviewed, across gender, used the HIV-positive status of their partners as a representation of their own HIV status. While they acknowledged the existence of discordance and the possibility of being in a discordant relationship, the chances were described as remote, in part because of the length of time they had lived with their spouses and the number of times they had unprotected sex with their partners. Testing for HIV was therefore viewed as unnecessary when the likelihood of being infected was already high. One study participant indicated that: *“I was sure that I was also going to get the same results. So there was no need for me to go for the test. You know we have had unprotected sex and one can get HIV through unprotected sex. Since my husband is HIV positive*, *I am also HIV-positive”* (20-year old woman). Similarly, a 47-year old man said: *“I also believe that I am HIV-positive because my wife has already tested HIV-positive”*.

#### Perceived psychological burden of living with HIV

While some study participants acknowledged that the HIV-positive status of their partners meant that they too could be infected, paradoxically, the perceived psychological burden of confirming that they also had HIV undermined the uptake of HIV testing. They preferred to live without knowing their HIV status. They perceived knowing one’s HIV status as having a deleterious mental health effect, which in turn was perceived as hastening deterioration of physical health. Some respondents narrated how their HIV-positive spouses had exhibited despair, became irritable, non-sociable and some adopted a fatalistic attitude towards life on account of their HIV-positive status. They worried that this could trigger adoption of similar behaviour if they sought HIV testing. When asked why he had not sought HIV testing, a 47-year old man explained that: *“When you are just feeling ok, to go and test and be found HIV-positive would just bring psychological problems. You start thinking too much about your health and your life and how your new status will change your life.”*

For some study participants, because HIV is not yet curable, knowing one’s HIV-positive status was synonymous with death itself. A 29-year old woman interviewed said: *“You know when you have HIV, even death comes to your door step because the disease cannot be cured. They say that there are drugs, but those ARVs do not cure HIV. So, it is better that I don’t know that I will die very soon.”* Paradoxically, this was the case despite the availability of life-prolonging ART and the acknowledgement that people on treatment were living longer and healthy lives.

#### Lack of self-efficacy

Since HIV testing is a pre-requisite for initiating HIV treatment, lack of self-efficacy, namely the inability to sustain positive treatment behaviour, dissuaded some study participants from testing. The lack of self-efficacy was attributed to three main reasons. First, some study participants felt that they would not manage to strictly adhere to the HIV treatment regimen, a requisite for achieving optimal viral suppression and living a normal and healthy life. Some narrated their experiences of failing to adhere to treatment for other health conditions such as malaria, which were short-term in nature, and therefore doubted their ability to stick to the HIV treatment regimen for the rest of their lives. They disliked the taste and smell of the drugs, a factor they said could impact on their ability to take their medication every day. One study participant explained: *“The problem with ARVs is that once you start taking ARVs, you take them for life. But herbs [alternative medication], you take when you feel like; and you can decide not to take for say 3 months. ARVs are very demanding”* (30 year old woman).

The influence of perceived inability to adhere to treatment on uptake of HIV testing was echoed by health workers. During the FGD, one of the male lay HCT counsellors said: *“Sometimes people worry about taking the drugs for the rest of their lives. So they fear to test because they are not ready to start treatment in case they are found HIV-positive.”*

Second, some study participants reported the challenges of integrating treatment into their day-to-day livelihood activities. This was particularly the case with respondents who were in self-employment, such as taxi drivers, construction workers and small-scale cross boarder traders, most of whom spent long hours away from their home. They feared that that they would default on accessing and taking their medication. As one 31-year old male study participant who worked as a taxi driver remarked: *“So, I thought that with my kind of work, because I work as a taxi driver, I wondered if I was going to manage to be driving if they say that those drugs make one feel drowsy. How am I going to manage to drive the car? You cannot be drowsy while driving, you will cause an accident, and I also cannot stop working because that is my only source of livelihood*.”

Thirdly, lack of self-efficacy was attributed to the side effects of HIV medication. The most common side effects mentioned were nausea, rash, feeling drowsy, changes in the texture of the skin and numbness, tingling or burning sensations. Fear of lipodystrophy - fat redistribution due to medication - was also a common theme. Asked why she had not sought HIV testing, a 29-year old female study participant answered: *“Me, I said, I will not go and test because I do not want to start ARVs. Because I have seen a lot of people taking ARVs face a lot of problems like having swollen legs, complaining of headache, they develop “ichifungalashi” (body numbness) that they cannot do anything.”* Local descriptions such as *‘kusintha kwa thupi’* (physical changes to the body), ‘*kuonongeka mawonekedwe’* (loss of physical appearance of the body) and*‘kutupa*’ (to look swollen) were widely used to describe the fear of these treatment-induced body changes. More so, observing the struggles of their spouses as they experienced and confronted treatment-related side effects did little to bolster their interest in and embolden them to seek HIV testing - the first step to get into treatment and care. This made them question the value of testing if they would not start treatment in the first place.

The negative attitude towards medication was reinforced by some deeply held community beliefs that HIV medication was insidiously harmful, and death of some PLHIV was attributed to the medication itself. A study participants whose aunt and two young sisters died shortly after being put on HIV medication attributed their death to the effects of the medication. A 42-year old male study participant explained: *“Then I also hear that those drugs destroy the liver, and that is why people keep on going to the clinic to check their liver. What kind of drugs are those? On one hand they say they prolong your life, but at the same time the same drugs are destroying your liver”*. A 30-year old female study participant revealed that a friend’s husband went blind after being started on treatment and *“He was told that the drugs were too strong and the veins collapsed.”*

#### Herbal remedies and non-HIV medication to mitigate HIV-related symptoms

As an alternative to HIV testing and starting treatment if found HIV-positive, all the spouses of PLHIV interviewed reported seeking recourse to alternative care - herbal remedies and conventional non-HIV medication to mitigate HIV-related symptoms. *Forever Aloe Vera gel -* a refined herbal product from South Africa - and *tembusha* (a local herbal plant), and a plethora of herbal remedies generally known as ‘immune boosters’ (including Chinese herbs) were the most widely used herbal remedies. The cost of these herbal remedies varied, with the most expensive being Aloe Vera gel, at US$15 per 1 l container. Due to high demand for herbal remedies, advertisements for different herbal products had become widespread; in the daily newspapers, through pamphlets handed out to motorists and pedestrians, posters stuck on trees and walls of private and public buildings.

Some participants also reported treating opportunistic infections using conventional non-HIV medication such as co-trimoxazole prophylaxis used by their HIV-positive marital partners. This in turn undermined uptake of HIV testing. Two study participants narrated this self-care practice. One said: *“I saw that people that have HIV but have not yet qualified to start ARVs take septrin. So, because I suspect that I have HIV, I just take septrin*….*Sometime back, when he (husband) was on septrin, we used to share the medication. So that is when I started organizing it on my own”* (45-year old woman). Another stated that: *“Even if I have not tested to confirm my HIV status, at least I take herbs in order to boost my immune system. At the moment, I am using tembusha. I make a 2.5 litre herbal solution and I take a glass in the morning, in the afternoon and in the evening”* (23-year old woman).

In addition, some study participants reported opting for faith healing instead of seeking HIV testing. This option has become common practice, in part, due to the growth of the evangelical Christian movement in Zambia. Some participants reported buying ‘anointing water’ - bottled water purported to have healing properties - from local churches and churches outside Zambia. Some ‘anointing water’ from Nigeria reportedly cost as much as US$100 for a 100 ml bottle. Others reported touching their television sets during Christian healing programmes on television in the hope of getting cured of HIV. Some acknowledged the power of prayer and faith in God in addressing health conditions, including incurable infections such as HIV. When asked about prayer and healing, one study participant acknowledged that: *“Yes, and it depends on your faith, like there is this friend of mine who went to [Dr*…*], she went there and she was prayed for*....*So people in the community would prefer going to churches to be prayed for than to come to the clinic*… *for HIV testing.”* (28-year old woman).

#### Self-stigma

Anticipated stigma - fear of ‘othering’, social isolation, gossip, labelling and public shaming - was not reported as a barrier to HIV testing. This was attributed to perceived low level of stigma in the community. This view was shaped by the perceived experiences of their HIV-positive partners. Instead, self-stigma (feeling of guilt and shame for having a stigmatising condition) and as already described above, moralisation and blaming behaviour within marital relationships, negatively affected uptake of HIV testing. A 31-year old man explained that: *“In the community there, stigma is no longer there. People would just sympathise with you. What is just remaining is self-stigma. You just feel guilty and ashamed that you have HIV. I avoid this feeling by not going for testing.”* This view was also shared by professional health care workers and lay HCT counsellors. When asked whether fear of enacted stigma prevented spouses of PLHIV from seeking HIV testing, a nurse based at the ART clinic disagreed that: *“No, stigma is very low…. You know some of our clients when they come here for treatment, at times their phones will ring and they would say ‘my friend, I am at the clinic, I am getting my ARVs.’ You know such statements and openness can give you an impression that people have now taken it [HIV] to be normal….What is killing people is the self-stigma that they have.”*

**Fig. 1:**
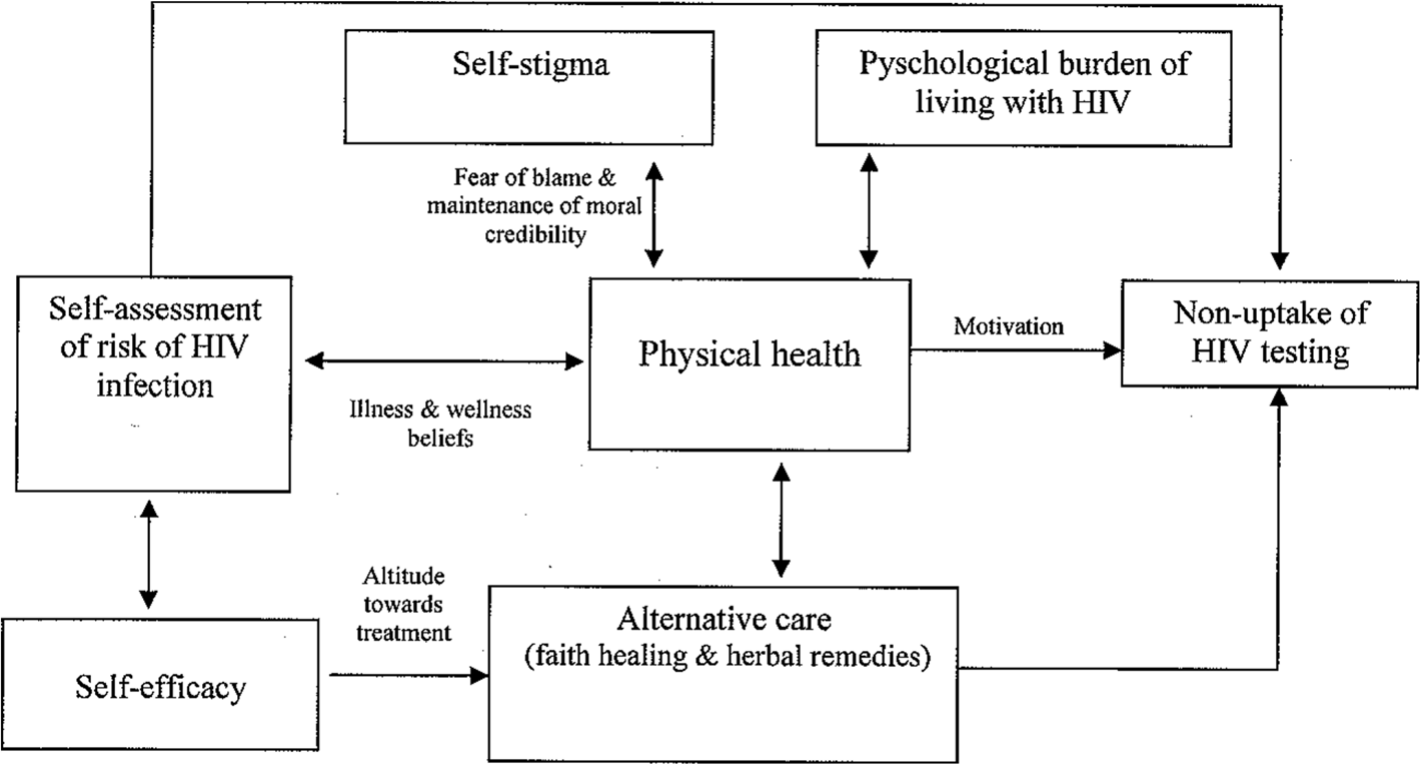
Conceptual model of relationship of barriers to uptake of HIV testing

The barriers to uptake of HIV testing described above are not mutually exclusive; they are interrelated and some coalesce to undermine HIV testing behaviour. For instance, while lay self-assessment of risk of HIV infection reduces motivation to test, perceived state of physical health, sometimes due to the use of herbal remedies and non-HIV conventional medication to mitigate HIV-related symptoms, inhibits uptake of HIV testing (Fig. 1). Similarly, lack of self-efficacy, which is sometimes driven by negative attitude towards HIV-medication, leads to uptake of alternative health care and this in turn dissuades people from seeking HIV testing. Also, while self-stigma, which manifests itself in desire to maintain moral credibility and absolve oneself from blame of HIV infection, and perceived psychological burden of living with HIV undermine uptake of HIV testing, these barriers may be modulated by the state of physical health, with study participants indicating willingness to test and initiate treatment if their health deteriorated and herbal remedies and conventional non-HIV medication were no longer effective in sustaining physical health (Fig. 1).

## Discussion

Our study found that five inextricably linked barriers explained why marital partners of PLHIV did not seek HIV testing despite knowing the HIV-positive status of their partners. These were: quest to maintain moral credibility, influenced by notion of masculinity and gendered power relationships; self-stigma; use of partner HIV status as a proxy for own HIV status; lay HIV treatment and health seeking practices; and lay self-assessment of wellness and illness. These are discussed in detail below. These findings corroborate previous studies and have implications for scaling-up HIV testing and treatment, a critical strategy for achieving the UNAIDS 90–90–90 treatment target-where 90 % of people living with HIV know their status; 90 % of people who know their HIV status are accessing treatment; and 90 % of people on treatment have suppressed viral loads [1].

### Maintenance of moral credibility, influenced by notion of masculinity and gendered power relationships

One of our findings was that non-uptake of HIV testing was done to maintain high moral standing within marital relationships. This was particularly so in distrustful relationships. For men, non-uptake of HIV testing was meant to avoid any admission of guilt for HIV infection, thereby maintaining moral credibility within the marital relationship. For women, non-uptake of HIV testing was intended to reflect being viewed as supporting and accepting the behaviour of the HIV-positive partner that could have led to HIV infection. These findings corroborate those of Larsson and colleagues [30] who have reported that unstable and distrustful nature of marital relationships undermined uptake of HIV testing. These findings have implications for HIV prevention. Given the fragility of marital relationships, strengthening the promotion of couple HIV testing is critical to mediate blaming attitude and may improve couples’ ability to manage HIV in marital relationships. This is especially so given the reported high rate of new infections in steady, long-term partnerships [31].

Relatedly, our findings suggest that the quest to maintain moral credibility in marital relationships is inextricably linked to the notion of masculinity - male notion of power, influence and being in control, and gender inequality - subordination of women in male–female relationships. For men, particularly when still physically healthy, non-uptake of HIV testing may therefore be used as a strategy to maintain social status, reputation and power as seeking HIV testing may be perceived as a sign of weakness which could consequently diminish influence, control and power over their marital partners. These findings corroborates other studies in Uganda [32], Zimbabwe [33] and Tanzania [34] which encapsulate how masculinity acts as a barrier to men’s use of HIV services. Siu and colleagues have described two different forms of masculinity – respectable masculinity which promotes positive health-seeking behaviour and reputation masculinity which undermines it [35]. In our study, reputation masculinity was found to be at play in influencing non-uptake of HIV testing among men. Thus, improving uptake of HIV testing by men requires encouraging the positive aspects of masculinity such as being socially and economically responsible and de-constructing the notion of reputation masculinity that undermine positive health-seeking behaviour, such as the sense of being strong and in control.

As a manifestation of gendered power relationships, for men, blaming women for HIV infection sometimes served other ends in relationships - to reinforce male control in marital relationships as exemplified by women’s fear that seeking HIV testing would lead to men shifting blame on them for HIV infection. A previous study in Uganda also reached this conclusion [36]. Similarly, Skovdal and colleagues [37] have reported that masculinity interferes with women’s uptake of HIV services. Our findings, therefore, call for the promotion of couple HIV testing to ensure joint knowledge and management of HIV status within marital relationships.

### Self-stigma

The impact of stigma on uptake of HIV testing has been reported in other studies [6, 11, 17, 32, 38]. In this study, fear of enacted stigma [39] was not found to be a barrier to HIV testing, even after probing for its influence. This was attributed to the experiences of study participants’ HIV-positive partners who were reported as not having experienced stigma. Instead, self-stigma was found to undermine uptake of HIV testing as reflected by the quest to avoid carrying the burden of shame associated with having HIV and moralising and blaming pattern described above. This finding about enacted stigma experiences should however be interpreted with caution. It might be limited to this particular group of marital partners of people living with HIV. Interviewing HIV-positive partners would have elicited insights into whether indeed they had not experienced stigma. Notwithstanding this limitation, counselling efforts aimed at creating social acceptance of HIV infection at individual level as well as in marital relationships are still warranted. The findings also echo the call by UNAIDS for all countries to implement the 2012 World Health Organisation (WHO) guidance on couples HIV testing and counselling in order to reach the targets set in the United Nations 2011 Political Declarations on AIDS [40]. As a caveat, couple HIV testing should be implemented with caution taking into account marital partners’ ‘lived’ experiences as couple HIV counselling and testing could aggravate already fragile marital relationships as previously reported in our study on experiences with couple testing in Zambia [41].

### Partner HIV status used as a proxy for own HIV status

The finding that marital partners use the HIV-positive status of their partners as a marker for HIV infection is consistent with evidence reported elsewhere [6, 42]. What this suggests is that while having an HIV-infected partner elevates the risk of HIV infection, this does not *ipso facto* lead to uptake of HIV testing, as those at risk assume that they are already infected. This is despite the existence of HIV discordance in marital relationships. This finding underscores a need for more HIV awareness campaigns on the existence and possibility of HIV discordance in marital relationships, and the importance of couple HIV counselling and testing to facilitate joint knowledge of HIV status and management of HIV condition. Optimising uptake of HIV testing also requires addressing the disjunction between perceived and actual risk of HIV infection as previously reported in other studies in Zambia [43], Malawi [44, 45] and Nigeria [46].

### Lay HIV treatment beliefs and health-seeking practices

Previous **s**tudies have reported how lack of self-efficacy dissuades individuals living with HIV from initiation of treatment [47–52]. Our findings suggest that lack of self-efficacy also extends to those who do not know their HIV status, thus inhibiting uptake of HIV testing. This is despite the widely reported positive impact of availability of ART on uptake of HIV testing [6, 12, 38, 53, 54]. Therefore, improving uptake of HIV testing also requires addressing lack of self-efficacy, which is in part influenced by negative HIV treatment and health-related beliefs. This finding should also be treated with caution. Given the rapid upswing in the numbers of PLHIV on treatment in Zambia since this study was carried out, these negative beliefs may have shifted with longer experience of ART. However, sensitisation campaigns against the perceived efficacy of herbal remedies and faith healing - which dissuade people from seeking HIV testing and initiate treatment - are still warranted given the reported widespread use of these practices.

The perception that treatment was insidiously harmful and had deleterious effects in the long-term was a recurring theme in this study, resulting in recourse to alternative forms of care - faith healing and herbal remedies - with the latter being reported as effective as antiretroviral treatment while hoping to get cured through the former. These treatment beliefs were couched in the experiences of patients started on early generation of antiretroviral treatment, whose adverse effects were reportedly severe and physically debilitating. What this suggests is that while a new generation of patient-tolerant antiretroviral drugs are now increasingly available, negative attitude towards treatment still persist. This does not only result in HIV-patient non-initiation of and attrition from antiretroviral treatment as reported in previous studies [52, 55]; it also undermines uptake of HIV testing - the first step in the ART care trajectory.

In Zambia, the fear of HIV medication needs to be contextualised. In 2007, the pharmaceutical company Roche announced that some batches of viracept (an ARV used in second-line treatment) had been accidentally contaminated with mesylate, which can cause cancer and genetic mutation [56]. While the drug was immediately discontinued, this created panic among people on treatment and reinforced the perception that HIV medication was harmful. Similarly, Zambia has progressively revised its treatment guidelines and the 2013 guidelines recommended the accelerated phasing out of stavudine (d4T) and zidovudine (AZT) in first-line combined antiretroviral therapy (cART) regimens and replacing them with efficacious drugs with better overall toxicity profile [57]. This was in line with the 2009 WHO recommendations to progressive phasing out d4T due to adverse effects such as disfiguring, unpleasant and potentially life threatening toxicity effects [58]. In our study, some respondents viewed the periodic changes to treatment guidelines and drug substitutions as emblematic of the dangers of the HIV medication, culminating in reluctance to seek HIV testing. Therefore, sensitisations to assuage these lay negative treatment beliefs and the availability of new patient-tolerant drugs as opposed to people seeking faith healing and scientifically unproven herbal remedies are warranted.

### Lay self-assessment of wellness and illness

We found that the overarching barrier to uptake of HIV testing was individuals’ perception of their corporeal health. Perceived good physical health dissuaded individuals from testing despite being at heightened risk of HIV infection. While study participants described a myriad of other factors inimical to uptake of HIV testing, and also reported resorting to alternative care, HIV testing was deferred until physical health had deteriorated. These findings are consistent with previous studies [9, 11, 38, 59]. This suggests that HIV testing is perceived narrowly as a gateway into treatment and care and not as a critical HIV prevention strategy. These findings corroborate those by Jürgensen and colleagues [6] who found that HIV testing was largely used as a diagnostic tool to access health care and not as an HIV prevention mechanism. Our findings suggest that the risk of declining health was counter-balanced by uptake of herbal remedies and conventional non-HIV medication to “boost” the immune system and to deal with episodic non-severe HIV-related symptoms. This undermined access to HIV testing and possible entry into ART care.

In view of the drive towards the ‘test and treat HIV’ model to HIV prevention, treatment and care as suggested by Granich and colleagues [60], our findings suggest that without addressing them, lay wellness and illness beliefs cast serious aspersions on the viability of universal HIV testing and immediate treatment as an HIV prevention strategy.

As a corollary, we found that fear of psychological burden associated with knowing one’s HIV status and its perceived negative impact on physical health dissuaded individuals from seeking HIV testing. This is consistent with previous studies [6, 13, 14, 38, 61, 62]. Our findings suggest that not seeking HIV testing despite acknowledging the possibility of being infected is used as a psychological buffer against the perceived mental burden of living with an incurable infection. This implies that despite the increasingly wider availability of life-saving ART, HIV still exudes fear of death in view of its incurable nature, and this fear may be exacerbated by the memories of suffering and death of people infected with HIV, including those on treatment. Thus, sensitization activities on the benefits of HIV testing regardless of physical health and addressing lay health beliefs that knowledge of HIV-positive status leads to rapid deterioration of health remain crucial in improving uptake of HIV testing.

The complex interplay of these findings makes two points saliently clear: an individual may not face a single barrier to uptake of HIV testing. Similarly, HIV testing behaviour is not a linear, sequential process - that knowing the HIV-positive status of a partner would *ipso facto* lead to uptake of HIV testing. These findings, therefore, point to a need for a multi-pronged, context-specific and individualised approach to addressing multiple, inter-linked and sometimes mutually reinforcing factors that undermine uptake of HIV testing.

## Limitations of the study

The study participants were recruited through their spouses receiving ART care at a local public sector clinic, and only those that agreed to be interviewed participated in the study. The findings may therefore not be representative of other individuals who refused to participate in the study. Consequently, this recruitment strategy could have led to some clustering of shared ideas and views. Interviewing the HIV-positive spouses could have provided more insights on non-testing behaviour through comparability of marital partners’ perspectives. Future studies should explore this further. A more general limitation concerns the generalisability of the findings. This study was conducted in a low-income setting with a small sample of respondents and aimed at gaining breadth and in-depth insights, in contrast to the purpose of quantitative research which aims to describe the frequency of such views. Therefore, similar studies are therefore warranted in other settings, including a larger, gender disaggregated sample size, for additional insights, such as the influence of other characteristics like age, duration of marital relationship and income status on uptake of HIV testing. Additionally, time has elapsed since the study was undertaken and since then, the number of PLHIV on treatment has greatly increased and alongside community experience of ART [63]. However, as our findings show, non-uptake of HIV testing still persists as demonstrated by some spouses who choose not to test for HIV despite knowing that the partner is living with HIV.

Notwithstanding the limitations, the strength of this study was the diverse representation of our study participants in terms of key demographic characteristics - age, gender and economic livelihood - and knowledge of treatment status of marital partner. Interviews with health care providers also provided additional insights, including for triangulation of data. The findings could apply to similar settings in urban areas in the country and provide useful insights that can inform policy and practice to improve uptake of HIV testing.

## Conclusions

HIV testing is an important strategy to combat HIV transmission and to facilitate entry into HIV care. However, our study has shown that knowing the HIV-positive status of a marital partner does not always lead to uptake of HIV testing. Instead, HIV testing behaviour is undermined by a complex and dynamic range of factors, which sometimes interact and coalesce. This study has found that individuals reach lay conclusions of already being infected on account of the HIV-positive status of their marital partners, thus viewing HIV testing as unnecessary. Testing is also not done to maintain moral credibility within marital relationship and to avoid legitimizing partner behaviour which could have led to HIV infection. Not knowing one’s HIV status is also aimed at creating a distance from HIV, thus acting as a buffer against the perceived psychological burden of living with HIV. While free HIV treatment has become widely available, its perceived negative effects and use of herbal remedies and conventional non-HIV medication to mitigate HIV-related symptoms and faith healing hamper uptake of testing. All these barriers appear to be modulated by the state of corporeal health, with individuals planning to test only after their health had deteriorated. Therefore, HIV care and prevention efforts should aim at addressing lay interpretations of risk of HIV infection, notion of masculinity, health and treatment beliefs, and promote the preventive and treatment benefits of early diagnosis of HIV. The reluctance by individuals to test despite knowing the HIV-positive status of their marital partners also calls for strengthening the promotion of couple HIV counselling and testing to ensure joint knowledge and management of HIV in marital relationships.

## Abbreviations

ART, antiretroviral therapy; DHS, Demographic and Health Survey; HCT, HIV counselling and testing; HIV, Human Immunodeficiency Virus; MCH, Maternal and Child Health; PLHIV, people living with HIV; SSA, sub-Saharan Africa; STI, sexually transmitted infections; TB, Tuberculosis; UNAIDS, Joint United Nations Programme on AIDS; WHO, World Health Organisation; ZEHRP, Zambia Emory HIV Research Project
